# Results from omic approaches in rat or mouse models exposed to inhaled crystalline silica: a systematic review

**DOI:** 10.1186/s12989-024-00573-x

**Published:** 2024-03-01

**Authors:** Laura Morin, Valérie Lecureur, Alain Lescoat

**Affiliations:** 1 Univ Rennes, CHU Rennes, INSERM, EHESP, IRSET (Institut de recherche en sante, environnement et travail), UMR_S 1085, 35000 Rennes, France; 2 Department of Internal Medicine, Rennes University Hospital, 35000 Rennes, France

**Keywords:** Crystalline silica, Environmental exposure, Omics, Mice, Rat, Autoimmunity

## Abstract

**Background:**

Crystalline silica (cSiO_2_) is a mineral found in rocks; workers from the construction or denim industries are particularly exposed to cSiO_2_ through inhalation. cSiO_2_ inhalation increases the risk of silicosis and systemic autoimmune diseases. Inhaled cSiO_2_ microparticles can reach the alveoli where they induce inflammation, cell death, auto-immunity and fibrosis but the specific molecular pathways involved in these cSiO_2_ effects remain unclear. This systematic review aims to provide a comprehensive state of the art on omic approaches and exposure models used to study the effects of inhaled cSiO_2_ in mice and rats and to highlight key results from omic data in rodents also validated in human.

**Methods:**

The protocol of systematic review follows PRISMA (Preferred Reporting Items for Systematic Reviews and Meta-Analyses) guidelines. Eligible articles were identified in PubMed, Embase and Web of Science. The search strategy included original articles published after 1990 and written in English which included mouse or rat models exposed to cSiO_2_ and utilized omic approaches to identify pathways modulated by cSiO_2_. Data were extracted and quality assessment was based on the SYRCLE’s Risk of Bias tool for animal studies.

**Results:**

Rats and male rodents were the more used models while female rodents and autoimmune prone models were less studied. Exposure of animals were both acute and chronic and the timing of outcome measurement through omics approaches were homogeneously distributed. Transcriptomic techniques were more commonly performed while proteomic, metabolomic and single-cell omic methods were less utilized. Immunity and inflammation were the main domains modified by cSiO_2_ exposure in lungs of mice and rats. Less than 20% of the results obtained in rodents were finally verified in humans.

**Conclusion:**

Omic technics offer new insights on the effects of cSiO_2_ exposure in mice and rats although the majority of data still need to be validated in humans. Autoimmune prone model should be better characterised and systemic effects of cSiO_2_ need to be further studied to better understand cSiO_2_-induced autoimmunity. Single-cell omics should be performed to inform on pathological processes induced by cSiO_2_ exposure.

**Supplementary Information:**

The online version contains supplementary material available at 10.1186/s12989-024-00573-x.

## Background

Crystalline silica (cSiO_2_) is a major component of rocks such as granite or sand which is found in materials used in the building industries such as cement or kitchen worktops. Workers from the construction or denim production industries as well as miners are particularly exposed to cSiO_2_ through inhalation [1]. Inhalation of cSiO_2_ increases the risk of respiratory disorders such as silicosis, but also increases the risk of systemic autoimmune diseases such as Systemic Sclerosis (SSc), Systemic Lupus Erythematosus (SLE) or Rheumatoid Arthritis (RA) [2–6].

Inhaled cSiO_2_ microparticles enter into the respiratory tract and can reach pulmonary alveoli [7]. The phagocytosis of cSiO_2_ particles by alveolar macrophages can induce cytotoxicity and macrophage cell death [8, 9] and can also initiate inflammatory responses and fibrosis through NLRP3 inflammasome activation [10]. Moreover, cSiO_2_-induced lung cell death is responsible for self-dsDNA release, STING-mediated sensing, IFN response and inflammation [11]. This process can be favoured by the impairment of efferocytosis capacities of macrophages exposed to cSiO_2_ [12]. cSiO_2_ is also known to induce systemic auto-immunity [13] but pathophysiological mechanisms involved in these effects remain unclear.

Omic methods are high-throughput technologies increasingly used in human and animal studies since the 1990s. They notably explore genomic, transcriptomic, proteomic or metabolomic data without a priori*.* They allow a better understanding of the overall biological processes and pathways involved in many disorders [14, 15] or in response to xenobiotic exposure including pollutants such as diesel exhaust particles [16]. Several studies using omic approaches have explored pathways involved in cSiO_2_ toxicity in mouse and rat models, but a comprehensive overview of these results is still lacking. Access to biological samples from patients exposed to cSiO_2_ is limited (limited access to bronchoalveolar lavages or lung biopsy) but rodent models can reflect cSiO_2_ exposure in human. Therefore, mechanisms identified in rodent models could also be relevant for humans [17]. A better identification of processes and pathways underlying cSiO_2_ toxicity in mouse or rat models may help design new therapeutic targets for cSiO_2_-related diseases in humans.

This systematic literature review (SLR) aimed at 1) providing a comprehensive state of the art on exposure methods and omic approaches used to study the effects of inhaled cSiO_2_ in mice and rats and 2) identify key results from omic data involved in cSiO_2-_related disorders and highlight those validated in human.

## Methods and analysis

The report for this SLR was designed in accordance with the PRISMA (Preferred Reporting Items for Systematic Reviews and Meta-Analyses) guidelines [18]. This protocol was registered in February 2022 on the International prospective register of systematic reviews Prospero (ID n°CRD42022299944) before research started.

### Search strategy

The search strategy aimed at selecting original studies mentioning cSiO_2_, mouse or rat models and at least one omic method, as summarised in the PECO form (see Additional file 1).

PubMed (Medline), Embase and Web of Science were selected for the identification of eligible titles and abstracts published before January 3, 2022. A specific search term strategy was designed for each database (see Additional file 2). Search equations were designed with the help of librarians. To test the relevance of our search strategies, eleven milestone articles considered as mandatory for our research question were selected a priori, based our knowledge from the field [19–29]. All eleven milestone articles were retrieved in each database confirming the relevance of the selected search terms. The first publications using omic techniques was published in the 90's, therefore only articles published after 1990 were explored. As this SLR focused on cSiO_2_, titles and abstracts mentioning nanoparticles of silica were excluded. Only rat or mouse experimentations were kept, others in vivo or in vitro models were excluded. Omic methods selected for this systematic review were genomics, transcriptomics, proteomics or metabolomics. Selected omic methods included RNA-seq, iTRAQ, Nanostring, microarray or mass spectrometry at the bulk or single-cell levels.

### Study selection

Title and abstract screening was performed using Rayyan software (https://www.rayyan.ai/). Two reviewers (AL & LM) independently screened all titles and abstracts, after publication and validation of the SLR protocol on Prospero. There were two screening phases for article selection. The first one consisted in screening titles and abstracts and the second selection consisted in screening selected full texts to include only relevant manuscripts for data extraction. A third reviewer (VL) resolved disagreements between reviewers for abstract and full text screening.

#### Title and abstract screening

Title and abstract screening aimed at pre-selecting articles that included mouse or rat models, silica exposure and the use of at least one omic method. Studies mentioning silica gel or column, silica-coated beads, silica nanoparticles or micro/nanospheres, mesoporous silica, silica spicules or bleomycin exposure were excluded. Studies without available English abstract were also excluded.

#### Full text screening

Inclusion and exclusion criteria for final article selection are summarized in Table 1. Briefly, only studies on mouse or rat models exposed to cSiO_2_ by inhalation were included. All strain, sex and age of mice and rats could be included except genetically modified animals such as knockout mice or rats because they were considered too different from the physiological conditions, and such results could not be applied to human physiology. NZBWF1 and NZM2410 were included because of their spontaneous genetic background for autoimmunity, similarly to what could be observed in human. All doses and frequency of exposure to cSiO_2_ were included. Only studies using omic approaches were finally kept for data extraction. Outcomes based on omic techniques were kept, including -but not limited to- genomic, transcriptomic, proteomic or metabolomic analyses; gene or protein expression profiles; miRNA-expression profiling/levels; RNA-seq; NanoString nCounter; iTRAQ; single-cell; microarray analyses; SAGE-seq; LC–MS. Outcomes assessed through other technological approaches than omic methods were excluded.

**Table 1:** Summary of inclusion and exclusion criteria for systematic review

	Inclusion criteria	Exclusion criteria
Article	Written in English and published after 1990	Review, systematic review, clinical trial, randomized controlled trial, meta-analysis and others articles that are not original studies were excluded
Experimental model	All strain, sex and age of mouse and rat models exposed to cSiO_2_ were accepted	In vitro, in silico or in vivo studies using models other than mouse and rat were excluded. Mouse and rat not exposed to cSiO_2_ particles were excluded. Mouse and rat genetically modified were excluded
Crystalline silica exposure	Only articles regarding silica in its crystalline form and cSiO_2_ exposure by inhalation were included. All doses and frequency of exposure were accepted	Articles mentioning silica gel or column, silica-coated beads, silica nanoparticles or micro/nanospheres, mesoporous silica, silica spicules or bleomycin exposure were excluded Studies using an exposure route other than inhalation were not considered
Outcomes	Outcomes demonstrated by the use of an omic approach on mouse or rat models exposed to cSiO_2_ by inhalation were kept, including but not limited to: genomic, transcriptomic, proteomic, metabolomic analysis; gene or protein expression profile; miRNA-expression profiling/level; RNA-seq; NanoString nCounter; iTRAQ; single-cell; microarray analysis; SAGE-seq; LC–MS	Outcomes demonstrated by other technological approaches than omic methods were excluded Omic approaches but not apply directly on mouse or rat models (in vitro omic approaches secondly confirmed by non-omic approaches in vivo) were also excluded
Comparator	Vehicle-treated control rat or mouse, controlled studies with a separate control group	Case studies, cross-over studies, studies without a separate control group were excluded

### Assessment of methodological quality

The quality of animal experiments was evaluated following the SYRCLE’s Risk of Bias (RoB) tool for animal studies [30]. This tool uses ten items to evaluate experimental bias and ten related questions. The response options are: “1 = yes” indicating that the study follows criteria of evaluation and it is free of bias; “0 = no” indicating the presence of bias in the study; “Un = unclear” indicating the absence of mention about these criteria in the study, with subsequent unclear risk of bias. The evaluation of quality assessment was performed by LM and checked by AL and VL.

### Data extraction

Data extraction was performed by LM and checked by AL and VL following a data extraction template that was adapted throughout the process. This template included the following items: 1. article characteristics including title, author, publication year; 2. Animal model including species, strain, age, sex, number of animals per group, control group considered as reference; 3. cSiO_2_ characteristics including size, purity and exposure including exposure route, dosage, frequency; 4. omic methods used, organ studied and outcomes including timing of measurement. Main biological processes, cellular components, molecular functions, pathways, networks and markers (gene, protein, miRNA) modulated by cSiO_2_ were extracted*.* Data were expressed in the format provided in the articles in accordance with existing databases for pathways, biological processes, networks and markers (Gene Ontology (GO), Kyoto encyclopaedia of genes and genomes (KEGG), etc.) and sub-classified depending on the assessment time: acute (≤ 1 week), sub-acute (2–11 weeks) and long-term effects (≥ 12 weeks) after cSiO_2_ exposure.

Data from a first set of 10% articles was also separately extracted by AL. Data independently retrieved by AL and LM were compared to ensure consistency and data extraction strategy was adapted in case of discrepancies. Once data extraction was completed by LM, accuracy of extracted data was checked by AL and VL for all articles.

## Results

### Studies included

**Fig. 1:**
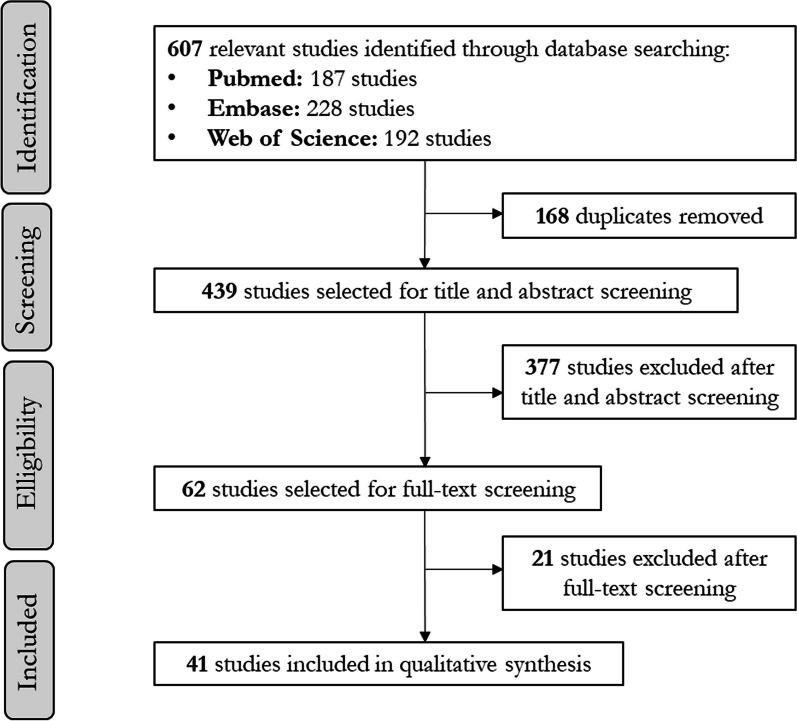
Process of inclusion and exclusion of studies

The flow chart of included studies is provided in Fig. 1. 607 relevant studies were identified through database searching. Among these studies, 439 were selected for title and abstract screening and 62 for full-text screening. There were 94.5% (N = 413/439) and 90.3% (N = 56/62) of agreement between AL and LM for title and abstract screening; and for full-text screening respectively. Based on full text evaluation, 41 studies were finally selected for data extraction [19–29, 31–59].

### Evaluation of experimental quality

The assessment of studies risk of bias is presented in additional file (see Additional file 3). All studies were free of selective outcome reporting (Q9) and 41.5% (N = 17) of the studies indicated an allocation sequence adequately generated (Q1) and similar animal groups at baseline (Q2). However, only 19.5% (N = 8) and 12.2% (N = 5) mentioned random outcome assessment (Q6) and blinding outcome assessor (Q7) respectively. Few studies mentioned random housing (Q4) and incomplete outcome data (Q8) (2.4% (N = 1) and 4.9% (N = 2) respectively). Any studies indicated suitable allocation concealment (Q3) and blinding caregivers or investigators (Q5).

### Rat and mouse characteristics

The characteristics of animal models used in included studies are described in Table 2. Rats were more frequently used than mice (N = 23 (56%)). The majority of animals was males (N = 30 (66.7%)) and their age at the beginning of experiment was comprised between 6 to 10 weeks (N = 25 (59.6%)). Some studies did not mention the sex (N = 2 (4.4%)) and the age (N = 1 (2.4%)) of animals. Few studies (N = 6 (14.6%)) used mouse strains prone for autoimmune diseases such as lupus-prone NZBWF1 and NZM2410. The number of animals per experimental group was commonly between 6 and 10 (N = 20 (46.5%)). However, the number of animals specifically studied with omic methods was lower than 5 in 38.6% (N = 17) of the studies and 31.8% (N = 14) of studies did not mention this number (N = 14 (31.8%)).

**Table 2:** Characteristics of animals used in omic approaches

Characteristics	N (%)
**Species**	
Rats	23 (56%)
Wistar	9 (22%)
Fisher (CDF strain)	7 (17.1%)
Sprague–Dawley	6 (14.6%)
Lewis	1 (2.4%)
Mice	18 (44%)
C57BL/6	11 (26.8%)
Lupus-prone NZBWF1	5 (12.2%)
NZM2410	1 (2.4%)
Balb/c	1 (2.4%)
**Sex**	
Male	30 (66.7%)
Female	13 (28.9%)
Not mentioned	2 (4.4%)
**Age at the beginning of experiment**	
≤ 6 weeks	8 (19%)
6–10 weeks	25 (59.6%)
≥ 11 weeks	7 (19%)
Not mentioned	1 (2.4%)
**Number of animals per group**	
Experimental group	
n ≤ 5	4 (9.3%)
6 > n > 10	20 (46.5%)
n ≥ 11	8 (18.6%)
Not mentioned	11 (25.6%)
Omic method group	
n ≤ 5	17 (38.6%)
6 > n > 10	13 (29.6%)
Not mentioned	14 (31.8%)

### Crystalline silica exposure

Characteristics of cSiO_2_ as well as the timing, dose and frequency of exposure are shown in Table 3. Half of the studies (N = 22 (53.6%)) exposed mice or rats to Min-U-Sil-5 silica, measuring 1.5–2 µm with a purity higher than 99% [60]. Other types of cSiO_2_ with same size and purity were used in several studies such as the silica from Sigma Aldrich mainly used on rat models (N = 10 (24.4%)) and Forsman Scientific only used on mouse models (N = 3 (7.3%)). Only one study exposed rats to DQ12, a silica with average size of 2.2 µm and purity of 87% [60]. Among the types of exposures, intratracheal instillation and inhalation chamber were the most chosen ones (N = 15 (36.6%) and N = 14 (34.1%) respectively), although inhalation chambers were only used on rats. Other methods included intranasal instillation (N = 8 (19.5%)) and oropharyngeal instillation (N = 4 (9.8%)) which were used only on mice.

**Table 3:** Characteristics of crystalline silica and exposure

Characteristics	N (%)	
Animal model	Mice	Rats
**Type**		
Min-U-Sil-5 (1.5–2 µm, > 99% purity)	12 (29.3%)	10 (24.4%)
Sigma Aldrich (1.5–2 µm, > 99% purity)	3 (7.3%)	10 (24.4%)
Forsman Scientific (1.6 µm, > 99% purity)	3 (7.3%)	–
DQ12 (2.2 µm, 87% purity)	–	1 (2.4%)
Not mentioned	–	2 (4.9%)
**Exposure method**		
Intratracheal instillation	6 (14.6%)	9 (22%)
Inhalation chamber	–	14 (34.1%)
Intranasal instillation	8 (19.5%)	–
Oropharyngeal aspiration	4 (9.8%)	–
**Dosage, frequency and duration of exposure**		
**Acute exposure**	14 (32.6%)	9 (20.9%)
1 dose	14 (32.6%)	8 (18.6%)
≤ 5 mg/exposure	9 (20.9%)	1 (2.3%)
6–20 mg/exposure	5 (11.6%)	–
≥ 21 mg/exposure	–	7 (16.3%)
1 dose during 6 h	–	1 (2.3%)
248 mg/m^3^	–	1 (2.3%)
**Chronic exposure**	6 (14%)	14 (32.6%)
2 doses	2 (4.7%)	–
2.5 mg at 3 days apart	1 (2.3%)	–
1 mg at 2 weeks apart	1 (2.3%)	–
3 doses	–	1 (2.3%)
45 mg at days 0, 7 and 14	–	1 (2.3%)
4 doses	4 (9.3%)	–
1 mg once weekly during 4 weeks	4 (9.3%)	–
3 h/day	–	4 (9.3%)
50 µg/m^3^ during 24 weeks	–	3 (7%)
50 µg/m^3^ during 4, 12, 16 or 24 weeks	–	1 (2.3%)
6 h/day	–	9 (20.9%)
≤ 2 mg/m^3^ during 5 days	–	1 (2.3%)
3–15 mg/m^3^ during 5 days or 3, 6, 12 weeks	–	8 (18.6%)
19 h/day	–	1 (2.3%)
1 mg/m^3^ during 2 weeks	–	1 (2.3%)
**Timing of outcome measurement**		
≤ 1 week	9 (14.1%)	8 (12.5%)
2–11 weeks	16 (25%)	11 (17.2%)
≥ 12 weeks	6 (9.4%)	14 (21.9%)

Half of studies exposed mice and rats to acute cSiO_2_ exposure (N = 23 (53.5%)) and the other half to chronic cSiO_2_-exposure (N = 20 (46.6%)). Mice were mainly exposed acutely to cSiO_2_ (N = 14 (32.6%)) while rats were mostly exposed chronically (N = 14 (32.6%)). Among studies using acute exposure (N = 23), the majority of animals were exposed to a single dose of cSiO_2_ (N = 22 (51.2%)) and the dosage was mainly lower than 5 mg for mice (N = 9 (20.9%)) and higher than 21 mg for rats (N = 7 (16.3%)).

Among chronic exposure, the daily cSiO_2_ exposure concerned only rat models. Twenty percent of the studies (N = 9) used chronic exposure on rats 6 h/day with dosage mainly comprised between 2 to 15 mg/m^3^ (N = 9) during several days (minimum of 5 days) or weeks (maximum of 12 weeks). The timing of outcome measurement was mostly comprised between 2 and 11 weeks for mice (N = 16 (25%)) and higher than 12 weeks for rats (N = 14 (21.9%)).

### Omic methods and organ studied

The summary of omic methods and organs or fluids used in studies is presented in additional file (see Additional file 4). The omic methods were more commonly applied to lung samples but blood, serum, plasma, kidney and spleen were also evaluated in some experiments. Sub-acute effects of cSiO_2_ exposure were more represented through omic methods (N = 30) than acute effects (N = 10) or long-term effects (N = 24), considering that one single study could use multiple omic approaches at the same time. All types of omics are provided in Table 4. This table highlights the diversity of techniques, particularly in transcriptomic and proteomic analyses, which can limit comparability among studies. Transcriptomic studies represented 76% (N = 35) of all omic methods used whereas proteomics represented 19.6% (N = 9) and metabolomics 4.3% (N = 2). mRNA microarray and mRNA-sequencing were the most commonly used transcriptomics methods (34.8% (N = 16) and 10.9% (N = 5) respectively). Among proteomic studies, mass spectrometry (N = 5 (10,9%)) and protein microarray (N = 4 (8,7%)) were mainly used.

**Table 4:** Types of omic methods used

Omic methods	N = 46 (100%)
**Genomics and transcriptomics**	**35 (76%)**
mRNA Microarray	16 (34.8%)
mRNA-seq	5 (10.9%)
NanoString nCounter	3 (6.5%)
miRNA-seq	2 (4.3%)
miRNA microarray	2 (4.3%)
lncRNA microarray	2 (4.3%)
lncRNA-seq	1 (2.2%)
3’ SAGE-seq	1 (2.2%)
Single cell RNA-seq	1 (2.2%)
SSH-cDNA sequencing	1 (2.2%)
NGS analysis	1 (2.2%)
**Proteomics**	**9 (19.6%)**
Mass spectrometry	5 (10.9%)
TMT LC–MS	1 (2.2%)
TOF–MS	1 (2.2%)
MALDI–TOF–MS PMF	1 (2.2%)
iTRAQ LC–MS	1 (2.2%)
MALDI-TOF–MS	1 (2.2%)
Protein microarray	4 (8.7%)
Cytokine microarray	1 (2.2%)
Protein microarray	1 (2.2%)
Autoantibody microarray	2 (4.3%)
**Metabolomics**	**2 (4.3%)**
NMR spectroscopy	1 (2.2%)
LC–MS	1 (2.2%)

Bold words representing the main technic used

As biological function is carried by proteins, we checked whether a change in mRNA expression was also related to a change at the protein level (Table 5). The number of transcriptomic studies for which mRNA results were validated at protein level represented 39.3% (N = 11) and the main techniques used were western blotting (14.3%, N = 4), ELISA/multiplex assays (21.4%, N = 6) and immunohistology (17.9%, N = 5).

**Table 5:** Validation of transcriptomic results at the protein level

Transcriptomic studies	N = 35 (100%)
Long non-coding RNA	**3 (8.6%)**
miRNA	**4 (11.4%)**
mRNA	**28 (80%)**
Validation at the protein level	11 (39.3%)
Western blotting	4 (14.3%)
ELISA/Multiplex assay	6 (21.4%)
Immunohistochemistry/immunofluorescence	5 (17.9%)

Bold words representing the main transcriptomic methods used

### Effects of crystalline silica as assessed by omic approaches in mouse

**Fig. 2:**
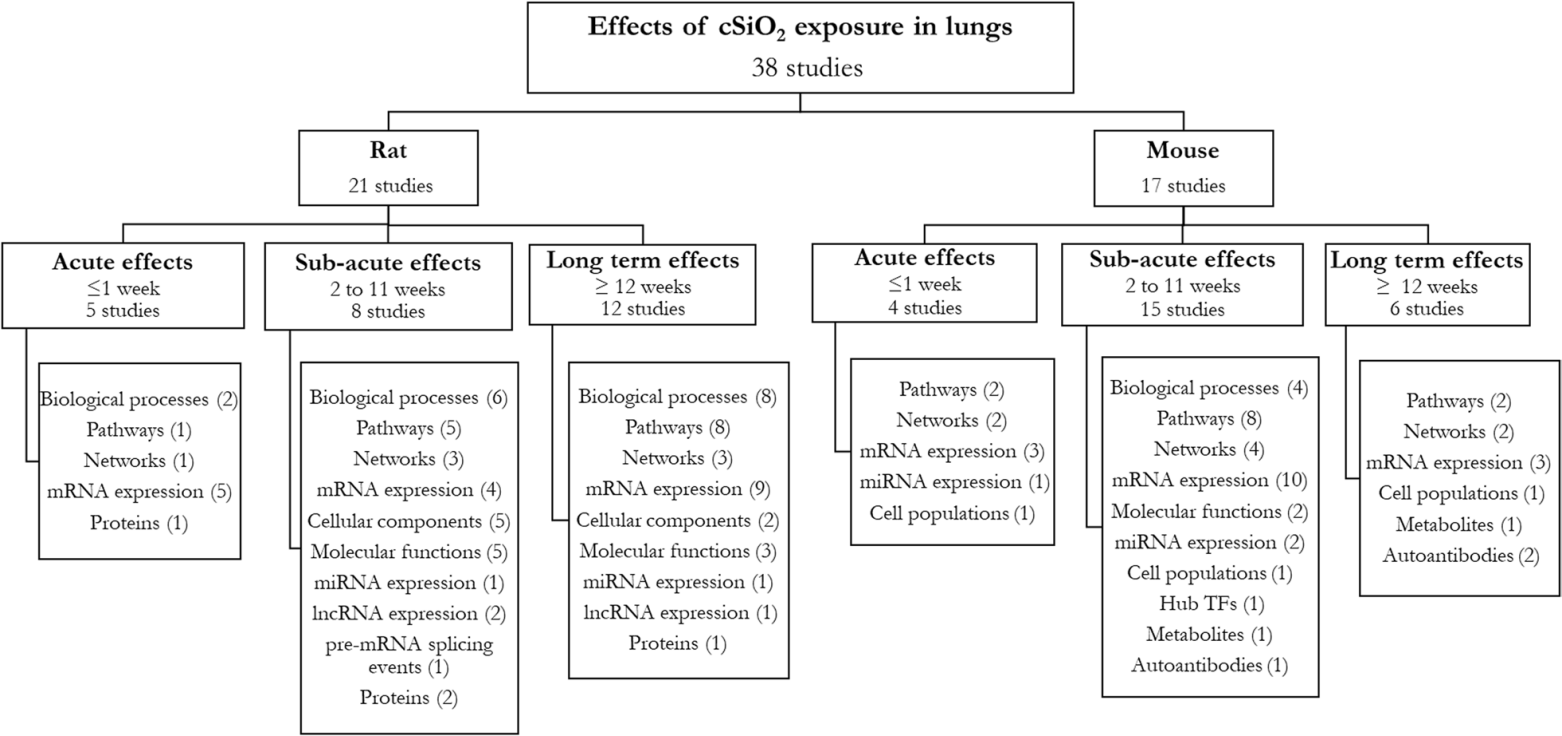
Crystalline silica exposure outcomes studied in rat and mouse lungs at several times

Lung was the most studied organ in rat and mouse models (N = 21/23 and N = 17/18 respectively) (see Additional file 5). Outcomes studied through omic methods after cSiO_2_ exposure at several time points in the lungs are shown in Fig. 2. Biological processes, pathways, networks and mRNA expression were the most frequently reported outcomes.

**Fig. 3:**
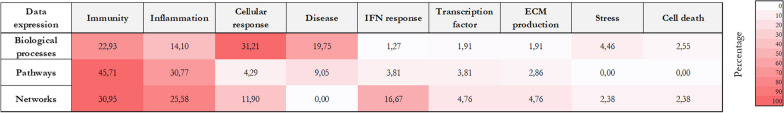
Heatmap representing the main domains of biological processes, pathways and networks found in included studies. Heatmap is expressed as the percentage of biological processes, pathways and networks of each domain mentioned in studies among the total number of biological processes (N = 157), pathways (N = 210) and networks (N = 42) retrieved in all included studies

**Fig. 4:**
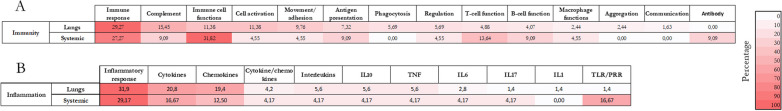
Heatmap representing the main immunity (**A**) and inflammation (**B**) sub-domains found in the lungs or at systemic level (serum, plasma, spleen) in included studies. Heatmap is expressed as the percentage of immunity (**A**) and inflammation (**B**) sub-domains of all biological processes, pathways and networks mentioned in studies among the total number of different immune responses or inflammation terms in lungs (N = 123 and N = 22 respectively) and at systemic level (N = 72 and N = 24 respectively) retrieved in all included studies

Heatmap representing the main domains of biological processes, pathways and networks found in omic approaches is presented in Fig. 3. Biological processes comprised mostly cellular and immunity domains while pathways and networks mostly included immunity and inflammation. The other domains were diseases, IFN response, transcription factor, ECM production, stress and cell death. Cellular response was the third most represented sub-domain in which movement, response to stimulus, adhesion, communication and growth/proliferation terms were mainly found (Additional file 6). Detailed characterisation of “Immunity” and “inflammation” in lungs or at systemic levels (serum, plasma, and spleen) is provided in Fig. 4A and B respectively. Several terms were common to the pulmonary and systemic levels such as those related to cytokines, notably IL-10, TNF, IL-6, IL-17 and IL-1 terms. In the lungs, several “immunity” and “inflammation” terms were linked to immune cell recruitment and activation such as cell activation, movement/adhesion, antigen presentation, aggregation, communication, cytokines, chemokines, B-cell, T-cell and macrophage functions. “Complement” term retrieved in the lungs could be linked to “phagocytosis” as complement-induced phagocytosis is a major innate immune mechanism. At systemic level (i.e. mostly blood and serum) in context of long-term cSiO_2_ exposure, adaptive immune response was frequently reported with the terms related to T- and B-cell function, antibody and antigen presentation.

### Validation of omic data in humans

Omic results obtained from animal studies provide data on gene and protein expression during exposure to cSiO_2_, however, these results need to be confirmed in humans to demonstrate their relevance for patients. Among the 41 included studies, only 17% (N = 7) validated their results from rodent in human samples (Table 6). The majority of these studies validated their results in seric or lung samples from patients with silicosis (N = 4). However, three studies validated their data in patients with idiopathic pulmonary fibrosis (IPF) which was less relevant to demonstrate the impact of cSiO_2_ exposure in human.

**Table 6:** Validation of results from mouse and rat models in humans

	OMIC methods used in rodent and main results	Type of silica exposure in rodents (acute or chronic) and time of outcome assessment (early or long-term outcome assessment)	Validation of omic results in rodent	Comparison of results in patient samples
Pang et al. [22]	LC–MS data shown higher levels of **TXA**_**2**_**, PGD**_**2**_ and PGE_2_ synthases in the silicosis mouse lungs compared to control mice	Acute exposure and long-term outcome assessment	Immunohistochemistry, qPCR and western blot allowed to validate the results for **TXA**_**2**_ and **PGD**_**2**_ but not for PGE_2_	The AA metabolic pathway was enriched in the lungs of silicosis patients using RNA-seq. The mRNA and protein expression levels of **PGD**_**2**_** and TXA**_**2**_ synthases were also increased in the lungs of silicosis patients compared with control (qPCR, western blot)
Shichino et al. [24]	3' SAGE-seq data has revealed gene modules, one upregulated and the other downregulated during fibrosis development in lung fibroblasts from mice exposed to cSiO_2_. The expression of the transcription factor **Srebf1** was downregulated during the lung fibrosis process	Acute exposure and long-term outcome assessment	ND	The gene set enrichment analysis (GSEA) of public transcriptome data from whole lungs or lung fibroblasts of human IPF patient showed that gene modules upregulated during fibrosis progression in mice were also enriched in human samples. However, gene modules downregulated in silicosis mice were more enriched in the lungs of healthy patients. The mRNA expression of **SREBF1** was downregulated in human lung fibroblasts derived from IPF patients
Gao et al. [40]	miRNA-seq identified downregulation of the **miR-411-3p** in the lungs of rat exposed to cSiO_2_	Chronic exposure and long-term outcome assessment	The **miR-411-3p** levels was lower in cSiO_2_-exposed rats lungs compared to control rats by in situ hybridization	The in situ hybridization technique showed lower levels of **miR-411-3p** in the lungs of silicosis patients
Ji et al. [42]	miRNA microarray has revealed a downregulation of **miR-486-5p** in cSiO_2_-exposed mice lungs	Acute exposure and long-term outcome assessment	The downregulation of **miR-486-5p** in cSiO_2_-exposed mice lungs is validated by qPCR	The **miR-486-5p** is downregulated in the serum from silicosis patients (qPCR). It was also downregulated in the lungs of IPF and silicosis patients (qPCR)
Cai et al. [20]	mRNA-seq analyses of lungs from rats exposed to cSiO_2_ shown an upregulation of **Spp1** expression, compared to non-exposed rats	Chronic exposure and long-term outcome assessment	Higher levels of **Spp1** proteins were shown in the cSiO_2_-exposed rat lungs by western blot and immunohistochemistry	**Spp1** protein levels were significantly higher in the serum from silicosis patients (ELISA) and its expression was associated with lung functions
Shichino et al. [56]	mRNA Microarray on CD45- cells isolated from cSiO_2_-exposed mice shown an upregulation of **Tnc, Mmp2, Grem1, Loxl2, Mmp14 and Thbs2** gene expression	Acute exposure and long-term outcome assessment	The upregulation of **Tnc, Mmp2, Grem1, Loxl2, Mmp14 and Thbs2** expression in CD45- cell from cSiO_2_-exposed mice was validated by qPCR	The analysis of data from public transcriptome data on IPF patient showed an upregulation of **Mmp2, Grem1, Mmp14, Thbs2, Loxl2, Tnc** gene expression in lung samples from IPF patient compared to healthy patient, that was similar to mice results
Koli et al. [45]	mRNA Microarray identified an upregulation of **Grem1** gene expression in the lung of cSiO_2_-exposed mice	Acute exposure and long-term outcome assessment	ND	The **Grem1** gene expression was higher in lung tissues and lung fibroblasts isolated from IPF patients (qPCR)

Pang et al. [22] and Shichino et al. [24] consistently showed changes in lipid metabolism in response to cSiO_2_ exposure in mice and confirmed these results in the lungs of patients with silicosis and/or IPF (Table 6). The arachidonic acid (AA) pathway was found activated and prostaglandin D2 (PGD2) and thromboxane A2 (TXA2) were upregulated in cSiO_2_-exposed mice and silicosis human lungs [22]. In addition, Srebf1, a transcription factor involved in the regulation of lipid metabolism, was downregulated in cSiO_2_-exposed mouse and the lungs of patients with IPF [24]. Gremlin1, an antagonist of bone morphogenetic protein (BMP), was found upregulated independently both in cSiO_2_-exposed mice and in the lungs of patients with IPF [45, 56], demonstrated the importance of this inhibitor as a key pro-fibrotic factor.

None of the omic studies exploring the systemic effects of cSiO_2_ in rodents validated their results in humans. This lack of data identifies a gap in the literature, with a need to fully validate systemic effects of cSiO_2_ and related systemic pathways.

## Discussion

This review provides a comprehensive state of the art on exposure methods and omic approaches used to study the effects of inhaled cSiO_2_ in mice and rats. Our work highlighted that male gender and rat models were more frequently used than females and mice and only a limited number of studies used mouse strains prone to autoimmunity, although it is a well endorsed effect of cSiO_2_ in human. Both acute and chronic exposure were explored in rats and mice and acute, sub-acute and long-term effects being mainly observed in the lungs. Transcriptomic approaches were more commonly performed, only a few studies used proteomic, metabolomic or single-cell omic approaches. Biological processes, pathways and networks were the most commonly reported outcomes in omic analyses. Among these outcomes, immunity and inflammation were the top two domains that were reported as impacted by exposure to cSiO_2_ in the lungs. Interestingly, only a few studies validate their transcriptomic results at the protein level in rodents and the number of translational studies was less than 20%, with only a few studies validating their results in humans.

### Experimental quality

The evaluation of experimental quality performed following SYRCLE’s risk of bias tool showed that studies quality was not sufficient in the majority of studies using omic approaches. Indeed, many criteria were insufficiently reported or lacking such as suitable allocation concealment, random housing, blinding caregivers or investigators and incomplete outcome. Experimental quality was not linked to the type of omic approaches. Improving experimental quality will be important in the future to allow a proper comparison of animal studies and to generalise their findings to clinical studies in humans.

### Species, sex and age of rodent models

The majority of animals used to study the effects of cSiO_2_ through omic methods were males. While transcriptomics, proteomics, genomics and single-cell omics were performed in males, only transcriptomic techniques were explored in female models. This focus on males in omic studies is relevant for human health as men are more represented among workers exposed to cSiO_2_ [1]. However, cSiO_2_ is also known to induce systemic autoimmunity which is over represented in women [61]. Using both male and female models in omic approaches is important considering the potential differential effects of cSiO_2_ on both genders [62]. As cSiO_2_ is able to promote autoimmunity [6], more studies should evaluate the effects of cSiO_2_ through omic approaches on mouse strains prone to autoimmunity to better understand the mechanisms involved in cSiO_2_-related autoimmune diseases.

The majority of experiments included young animals with a maximal age of sixteen weeks at the beginning of experiment. This could be considered as selection and experimental bias, considering that cSiO_2_ effects on old-age models could especially be relevant as a role of senescent cells has recently been suggested in silica-induced pulmonary fibrosis [63].

### Types of crystalline silica

Quartz is the most abundant form of cSiO_2_ in nature and is used as a raw material in several industrial and building processes. Several quartzes were used in experimental studies such as Min-U-Sil or DQ12. Min-U-Sil 5 is composed of 99% of quartz while DQ12 is a quartz sand composed of 87% of cSiO_2_ and 13% of amorphous silica and kaolinite, their particle size is less than 5 µm [60]. A large number of studies included in this SLR exposed mice and rats to Min-U-Sil5 or to other types of silica with the same characteristics (Sigma Aldrich, Forsman Scientific). However, the global effects of exposure to artificial stone, containing silica and resins, remains to be explored whereas an epidemic of silicosis was recently observed in artificial stone producer countries [64].

### Dosage and frequency of cSiO_2_ exposure

Both acute and chronic exposure to cSiO_2_ were evaluated through omic techniques in rats and mice. Chronic exposure is relevant to translate the effects of cSiO_2_ in mice and rats to human workers but acute exposure can be representative of acute silica hazards such as silicoproteinosis [65]. Based on the limitation established for workers exposure, it is determined that 8.28 mg of inhaled cSiO_2_ in mice corresponds to an exposure of human workers during 40 years [4]. Among acute exposure, the most commonly used dosages in omic studies were less than 5 mg per exposure for mice corresponding to less than one half of human lifetime exposure. Dosage of cSiO_2_ for rat acute exposure were mostly higher than 21 mg, which is consistent with the higher weight of the rats. Chronic exposures carried-out in most omic studies consisted in exposing rats to 2 to 15 mg/m^3^ of cSiO_2_ 6 h per day during several days or weeks that is more representative of human workers chronic exposure. Transcriptomic and proteomic techniques were well distributed in the different dosage and frequency of exposure.

Timing of outcome assessment after cSiO_2_ exposure is crucial to identify relevant effects. Outcome measurements in omic studies mainly concerned sub-acute effects of cSiO_2_ but chronic effects were also well studied with transcriptomic and proteomic approaches. Silicosis is induced by cSiO_2_ exposure and is characterised by chronic inflammation and fibrosis [66]. As the onset of symptoms classically occurs at a later stage of the disease, sub-acute and long-term effects of cSiO_2_ are especially relevant to study such late outcomes. Moreover, long-term effects allow the exploration of autoimmune features such as cSiO_2_-related autoantibody production [13].

### Omic methods

Omic approaches such as genomics, transcriptomics, metabolomics or proteomics provide a global perspective from large dataset in organisms and contribute to the understanding of mechanisms involved in human diseases. There are various technologies to study omics but, separately, they cannot explore the entire complexity of organisms and each of these techniques has their strengths and limitations.

Transcriptomic studies were the most omic approaches used in included studies and microarray analysis was more used than RNA-seq. Microarray allows the identification of differentially expressed genes using chip with thousands of short single-stranded DNA sequences, therefore there is an a priori knowledge of the sequences and their number is limited. On the other hand, RNA-seq allows sequencing whole transcript of cells or tissues without quantitative limitations and a priori. Studies using RNA-seq were published after 2018 while those using microarray were published earlier corresponding to the development and wider use of RNA-seq characterised by a higher sensitivity and specificity as well as broader data sets with higher comprehensiveness.

Although differentially expressed genes allows a better understanding of the mechanisms involved, it is essential to focus on the proteins encoded by these differentially expressed mRNAs as proteins are the actual actors of the considered physiological and pathological processes. Despite such important considerations, only less than a half of transcriptomic studies validated their results at the protein level. Moreover, proteomic approaches were not widely used in included studies and only a few studies used mass spectrometry techniques and microarrays to identify differentially expressed proteins. Mass spectrometry enables identification and quantification of whole differentially expressed proteins without targeted strategy while protein microarrays require target proteins. Autoantibodies microarray development could be of great interest to profile circulating autoantibodies in autoimmune diseases [67] and, systematically apply such technics in studies assessing the effects of cSiO_2_ on adaptive immunity may help decipher the key processes involved in cSiO_2_-related autoimmunity.

Transcriptomics and proteomics provide data on differentially expressed genes and proteins respectively but do not provide direct results regarding cells involved in the process at stake. For this reason, single-cell omics have been developed and single-cell transcriptomics (single-cell RNA-seq), proteomics (mass cytometry CyTOF) or spatial omics (Hyperion) are increasingly used [68]. However, single-cell techniques were rarely found in included studies, only one study published in 2021 used single-cell RNA-seq [25]. We may expect that the high dimensional single cell analysis at the protein level might be more used to identify which cell types can drive cSiO_2_ effects. Multi-omic approaches combining transcriptomics, proteomics and metabolomics in a same study and/or protocol may also help improve the overall understanding of the mechanisms involved in the physiopathology of cSiO_2_ and foster the design of new therapeutic targets [14].

### cSiO_2_ exposure effects

In included studies, omic approaches were mainly carried out on lungs and more rarely on blood, plasma, serum, spleen and kidney. However this focus on lungs may lead to a gap of knowledge regarding the systemic effects of silica dust [69]. Biological processes, pathways, networks and mRNA expression were the most studied omic outcomes in response to cSiO_2_ exposure in rodents. Among them, immunity- and inflammation-related outcomes were the two most frequent domains reported both at the lung and systemic level, suggesting a similar and global response to cSiO_2_ exposure. Indeed, a wide range of cytokines were retrieved in response to cSiO_2_. The identification of IL-1β is consistent with the well-described inflammasome (NLRP3) activation induced in response to cSiO_2_, along with the involvement of TLR pathway resulting in IL-1β cytokine release [70]. Moreover, the cSiO_2_-induced IL-17 pathways identified in included studies was previously reported [71]. This interleukin is known to be increase in autoimmune disorders [72] and could be implicated in cSiO_2_-induced autoimmunity [13]. Innate and adaptive immunity both play a role in the response to cSiO_2_ exposure. Indeed, terms related to cell activation, functions and recruitment were mainly found at pulmonary and systemic levels, which is consistent with the known recruitment and activation of inflammatory cells in response to cSiO_2_. Phagocytosis were also retrieved in lungs, consistently with existing data regarding the response to cSiO_2_ exposure [9]. Identification of the complement term was in accordance with its role in silicosis and inflammation [73]. The adaptive immunity-related terms such as antibody and T- and B-cell function were especially identified as long-term effects and at systemic levels, that is in coherence with the time of an autoimmune response to cSiO_2_ [13, 17]. All these results from omics studies on rodents enable us to appreciate the overall effects of cSiO_2_ exposure in rodents, which are now well described in the literature. Therefore, it would be interesting to investigate the mechanisms involved in its effects and to carry out translational studies to gain a better understanding of the effects of crystalline silica on human pathologies.

### Translation of omic results from rodents to humans

Validating results obtained in murine models into humans is an important step in determining whether the results from animal studies are relevant to patients. Among included studies, less than 20% validated in humans the omic results obtained in rodents. More translational studies are thus needed. Indeed, rodent models are required to explore the effects of cSiO_2_ since doses and frequencies of exposure can be controlled, making it easier to obtain reproducible and comparable results among studies. However, confirming these results in humans by studying organs, peripheral blood mononuclear cells or serum is still mandatory. Regarding systemic effects of cSiO_2_, none of the included studied validate their results in human. This could be explained by the lack of available human samples, suggesting that fostering the implementation of biorepositories (PBMC, serum and/or plasma biobank) is an important unmet need. Beyond biorepositories, public transcriptome data in humans are available through open access and could enable in silico comparisons with animal results.

Validated results in patients with silicosis or IPF revealed changes in lipid metabolism in response to cSiO_2_ exposure in two independent studies [22, 24]. The AA pathway was found reprogrammed, with an up-regulation of PGD2 and TXA2, two inflammatory mediators potentially involved in silicosis-related fibrogenesis. Such effects may rely on Srebf1, a transcription factor involved in the regulation of AA pathways, that was found downregulated in one of the included study [74, 75]. Moreover, transcriptome network analyses shown that Srebf1 was connected with some pro-fibrotic gene such as Gremlin1 [24], that was shown upregulated in two independent studies first in cSiO_2_-exposed mice (long-term effect) and then in lung of patients with IPF [45, 56]. The overexpression of this protein, as an antagonist of BMP, can imbalance the BMP and TGF-ꞵ pathways, leading to fibrosis [76]. Gremlin1 was also upregulated in response to asbestos in the lungs of exposed-mice and to coal dust inhalation in the serum of patient with coal worker’s pneumoconiosis [77, 78]. It was also retrieved at high levels in the serum of SSc patients with interstitial lung disease (ILD), an autoimmune disease for which exposure to cSiO_2_ is a risk factor [79]. Therefore, Gremlin1 could be use as biomarker of asbestos, coal or cSiO_2_ dust exposure.

### Perspectives for omic methods to study cSiO_2_ effects

This comprehensive overview on cSiO_2_ effects highlighted gaps in the literature. 1/ cSiO_2_ effects on male mouse or rat models are well studied through omic approaches but analysis on female models only focused on transcriptomic approaches and need to be extended. 2/ Min-U-Sil silica is more commonly cSiO_2_ type used in omic studies. The comparison between Min-U-sil, DQ12 or other type of cSiO_2_ exposure through omic methods may help identify key differences in the biological impact of different types of silica. 3/ Transcriptomic studies need to be better validated at protein level and/or in tissues, since proteins are the actual players of the biological response. 4/ Systemic effects of cSiO_2_ should be further studied and omic techniques should be performed on other samples than lungs such as whole blood, kidney or spleen; notably to explore the autoimmune effects of silica [17]. Moreover, only a few omic analyses have been performed on autoimmune disease prone models exposed to cSiO_2_ despite the lack of understanding of cSiO_2_-induced autoimmunity. 5/ The use of single-cell transcriptomic and proteomic analyses that provide data on each cell type should also be fostered as they allow the identification of specific cell types involved in cSiO_2_-related pathological processes. 6/ Among included studies using omic approaches, only a few studies have compared omic results from rodents to omic data from human cohorts exposed to cSiO_2_ although such translational approaches are crucial to confirm that biological processes, pathways or networks identified in rodents are also relevant in humans. Translational studies are therefore still needed.

### Strengths and limitations

Strengths of the proposal: This SLR is the first study providing a comprehensive and systematic overview of studies exploring the effects of inhaled cSiO_2_ in the mouse or rat models using omic approaches. Our SLR follows the PRISMA recommendations for SLRs [18]. We used three different databases for article selection providing a comprehensive analysis of the literature on the subject. Moreover, the protocol of this systematic review was published on Prospero prior to the beginning of abstract screening. Combining transcriptomic and proteomic results provide an unprecedented overview of the effects of inhaled cSiO_2_ in mouse and rat models. This review includes studies using different time points of outcome measurement after the last cSiO_2_ exposure, at both pulmonary and systemic levels.

Limitation of the proposal: we used an a priori definition of omic approaches that is not endorsed or validated. The heterogeneity of the protocols used in the studies (dosage, frequency, duration, cSiO_2_ type and methods of exposure) can be considered a limitation as it may preclude a direct comparison of the obtained results. Results may also vary depending on the omic techniques, the platform where the omic approaches are performed and statistical analyses of the data. In addition, we did not specifically explore the impact of the dose or frequency of cSiO_2_ exposure. In our work, we only explored the main biological processes, pathways and networks identified in the studies, some pathways are therefore not retained although they may have a role in cSiO_2_ effects. By focusing on studies using omic techniques, this review may not include results obtained from others techniques and therefore all the mechanisms modulated by cSiO_2_ could not be identified although such aim was beyond the scope of this study.

## Conclusion

In this SLR review, providing an overview of cSiO_2_ effects in mice and rats, omic techniques were more commonly carried out on lungs and analysis of the systemic effects of cSiO_2_ were neglected. Perform further omic analysis on autoimmune prone mouse models and on female models may help identify mechanisms involved in cSiO_2_-induced autoimmunity. Proteomics and single-cell analysis are still lacking to identify the main actors of the pathological processes induced by cSiO_2_ and validation in humans are only performed in less than 20% of the available studies. Current validated results from independent omic studies in rodent translated to humans, supported the impact of cSiO_2_ on lipid metabolism (AA pathways) and the role of Gremlin1 as a TGF-ꞵ regulator in cSiO_2_-related fibrogenesis. Identifying and validating new prominent pathways could help design and evaluate relevant therapeutic approaches for lung and systemic effects of cSiO_2_.

### Supplementary Information

**Additional file 1**. **Table S1.** The PECO (Population, Exposure, Comparator, Outcome).

**Additional file 2.** **Table S2.** Search terms used in databases.

**Additional file 3.** **Table S3. **Assessment of studies risk of bias using SYRCLE’s risk of bias tool.

**Additional file 4.** **Table S4.** Summary of omics methods and organs used in studies

**Additional file 5.** **Fig. S1.** Crystalline silica exposure outcomes studied in rat and mouse organs

**Additional file 6.** **Fig. S2.** Heatmap representing the main cellular responses sub-domains found in the lungs or at systemic level (serum, plasma, spleen) in included studies. Heatmap is expressed as the percentage of cellular response sub-domains of all biological processes, pathways and networks mentioned in studies among. the total number of different cellular response terms in lungs (N = 61) and at systemic level (N = 2) retrieved in all included studies.

## Data Availability

Not applicable.

## Abbreviations

AA: Arachidonic pathway
BMP: Bone morphogenic protein
cSiO_2_: Crystalline silicon dioxide
CyTOF: Cytometry by time of flight
DNA: Deoxyribonucleic acid
ECM: Extracellular matrix
ELISA: Enzyme-Linked Immunosorbent Assay
GO: Gene ontology
IL: Interleukin
IFN: Interferon
lncRNA: Long non-coding RNA
IPF: Idiopathic pulmonary fibrosis
iTRAQ: Isobaric tags for relative and absolute quantitation
KEGG: Kyoto encyclopaedia of genes and genomes
LC-MS: Liquid chromatography-mass spectrometry
MALDI-TOF-MS PMF: Matrix-assisted laser desorption/ionization-time of flight-mass spectrometry-peptide mass fingerprint
Mesh: Medical subject headings
miRNA: Micro RNA
mRNA: Messenger
ND: Not determined
NF-kB: Nuclear factor-kappa B
NGS: Next-generation sequencing
NLRP3: NOD-like receptor family, pyrin domain containing 3
NMR: Nuclear magnetic resonance
NZBWF1: New Zealand Black and New Zealand White F1
NZM2410: New Zealand Mixed 21410
PBMC: Peripheral blood mononuclear cell
PECO: Population, Exposure, Comparator, and Outcome
PGD2: Prostaglandin D2
PRISMA: Preferred Reporting Items for Systematic Reviews and Meta-Analyses
qPCR: Quantitative polymerase chain reaction
RA: Rheumatoid arthritis
RNA: Ribonucleic acid
RNA-seq: RNA sequencing
RoB: Risk of Bias
SAGE-seq: Serial analysis of gene expression sequencing
SLE: Systemic lupus erythematosus
SLR: Systematic Literature Review
Srebf1: Sterol regulatory element binding transcription factor 1
SSc: Systemic sclerosis
SSH-cDNA: Suppression subtractive hybridization complementary DNA
STING: Stimulator of Interferon genes
SYRCLE: Systematic Review Center for Laboratory Animal Experimentation
TGF-ꞵ: Transforming growth factor beta
TLR: Toll-like receptors
TMT LC-MS: Tandem mass tag liquid chromatography-mass spectrometry
TNF: Tumor necrosis factor
TOF-MS: Time of flight mass spectrometry
TXA: Thromboxane A2
