# Supplementary material for: Results from omic approaches in rat or mouse models exposed to inhaled crystalline silica: a systematic review
Source: Part Fibre Toxicol. 2024 Mar 1;21:10. doi: 10.1186/s12989-024-00573-x (PMC10905840; doi:10.1186/s12989-024-00573-x)
Supplement: Supplementary file 1 — Additional file 1. Table S1. The PECO (Population, Exposure, Comparator, Outcome). [file 12989_2024_573_MOESM1_ESM.docx]

| Supplementary Table 1: The PECO (Population, Exposure, Comparator, Outcome) | |
| --- | --- |
| Variable | **Description** |
| Population | Mouse and rat models |
| Exposure | Any dose of inhaled cSiO_2_ |
| Comparator | Rats or mice not exposed to cSiO_2_ |
| Outcome | Genomics, transcriptomics, proteomics and metabolomics ouctomes (biological processes, pathways, networks) |
