# Supplementary material for: Results from omic approaches in rat or mouse models exposed to inhaled crystalline silica: a systematic review
Source: Part Fibre Toxicol. 2024 Mar 1;21:10. doi: 10.1186/s12989-024-00573-x (PMC10905840; doi:10.1186/s12989-024-00573-x)
Supplement: Supplementary file 2 — Additional file 2. Table S2. Search terms used in databases. [file 12989_2024_573_MOESM2_ESM.docx]

| Supplementary Table 2: Search terms used in databases | |
| --- | --- |
| Database | **Search terms** |
| Pubmed | ("Silicon Dioxide"[Mesh] OR "Quartz"[Mesh] OR "Silicosis"[Mesh] OR "Idiopathic Pulmonary Fibrosis/chemically induced"[Mesh] OR Silica or Silicosis) NOT Nanoparticle AND ("Rats"[Mesh] OR "Mice"[Mesh] or Mouse or Rats) AND ("Transcriptome"[Mesh] OR "Gene expression profiling"[Mesh] OR "MicroRNAs/genetics"[Mesh] OR "RNA-Seq"[Mesh] OR "Microarray Analysis"[Mesh] OR "Proteome"[Mesh] OR "Proteomics"[Mesh] OR "Metabolome"[Mesh] OR "Metabolomics"[Mesh] OR "Single-Cell Analysis"[Mesh] OR "Nanostring" OR "Sequence Analysis, RNA"[Mesh] or Transcriptome) |
| Embase | ('silicon dioxide' OR 'silicosis' OR 'crystalline silica' OR quartz) NOT 'nanoparticle' AND ('rat' OR 'mouse' OR 'murine') AND ('transcriptome' OR 'transcriptomics' OR 'gene expression profiling' OR 'sequence analysis' OR 'rna sequencing' OR 'proteome' OR 'proteomics' OR 'microarray' OR 'microrna' OR 'multiomics' OR 'itraq' OR 'nanostring' OR 'single cell' OR 'metabolome' OR 'metabolomics') |
| Web of Science | TS=(Silica OR silicon dioxide OR silicosis OR quartz) AND TS=(Transcriptome OR transcriptomic OR gene expression profiling OR sequence analysis OR RNA-seq OR proteome OR proteomic OR microRNA OR microarray OR multi-omics OR transcriptomic OR iTRAQ OR nanostring OR single-cell OR metabolome OR metabolomic) AND TS=(mouse OR rat ) NOT TS=(nanoparticle*) |
