# Supplementary material for: Results from omic approaches in rat or mouse models exposed to inhaled crystalline silica: a systematic review
Source: Part Fibre Toxicol. 2024 Mar 1;21:10. doi: 10.1186/s12989-024-00573-x (PMC10905840; doi:10.1186/s12989-024-00573-x)
Supplement: Supplementary file 3 — Additional file 3. Table S3. Assessment of studies risk of bias using SYRCLE’s risk of bias tool. [file 12989_2024_573_MOESM3_ESM.docx]

| **Supplementary Table 3:** Assessment of studies risk of bias using SYRCLE’s risk of bias tool | | | | | | | | | | |
| --- | --- | --- | --- | --- | --- | --- | --- | --- | --- | --- |
| **Studies** | **Q1: Sequence generation** | **Q2: Baseline similarities** | **Q3: Allocation concealment** | **Q4: Random housing** | **Q5: Blinding caregivers and/or investigators** | **Q6: Random outcome assessment** | **Q7: Blinding outcome assessor** | **Q8: Incomplete outcome data** | **Q9: Allocation sequence** | **Q10: Other bias** |
| Bates et al., 2018 | 1 | U | U | U | U | U | U | 1 | 1 | U |
| Beamer et al., 2010 | U | U | U | U | U | 1 | U | U | 1 | U |
| Benninghoff et al., 2019 | U | U | U | U | U | U | U | U | 1 | U |
| Bo et al., 2020 | 1 | U | U | U | U | U | U | 0 | 1 | U |
| Brown et al., 2005 | U | U | U | U | U | U | 1 | U | 1 | U |
| Cai et al., 2020 | 1 | 1 | U | U | U | U | U | U | 1 | U |
| Cai et al., 2021 | 1 | 1 | U | U | U | U | U | 1 | 1 | U |
| Cao et al., 2021 | 1 | 1 | U | U | U | U | U | U | 1 | U |
| Chauhan et al., 2021 | U | U | U | U | U | U | U | U | 1 | U |
| Chen et al., 2018 | U | U | U | U | U | U | 1 | U | 1 | U |
| Dorman et al., 2012 | 1 | 1 | U | U | 0 | 1 | U | U | 1 | U |
| Ellinger-Ziegelbauer et al., 2009 | 1 | U | U | U | U | U | U | U | 1 | U |
| Faxuan et al., 2012 | 1 | 1 | U | U | U | U | U | U | 1 | U |
| Gao et al., 2020 | U | 1 | U | U | U | U | U | U | 1 | U |
| Hu et al., 2008 | 1 | U | U | U | U | U | U | U | 1 | U |
| Jin et al., 2008 | 1 | 1 | U | U | U | 1 | U | U | 1 | U |
| Ji et al., 2015 | U | 1 | U | U | U | U | U | U | 1 | U |
| Kim et al., 2005 | U | U | U | U | U | U | U | U | 1 | U |
| Koli et al., 2016 | U | U | U | U | U | U | 1 | U | 1 | U |
| Langley et al., 2011 | U | 1 | U | U | U | U | U | U | 1 | U |
| Pang et al., 2021 | 1 | 1 | U | 1 | U | 1 | U | U | 1 | U |
| Pestka et al., 2021 | 1 | 1 | U | U | U | 1 | 1 | U | 1 | U |
| Rajasinghe et al., 2020 | U | U | U | U | U | U | U | U | 1 | U |
| Sager et al., 2020 | U | U | U | U | U | U | U | U | 1 | U |
| Sai et al., 2019 | 1 | U | U | U | U | 1 | U | 0 | 1 | U |
| Sai et al., 2021 | 1 | 1 | U | U | U | U | U | U | 1 | U |
| Sellamuthu et al., 2011 | U | U | U | U | U | U | U | U | 1 | U |
| Sellamuthu et al., 2012 | U | U | U | U | U | U | U | U | 1 | U |
| Sellamuthu et al., 2011 | U | U | U | U | U | U | U | U | 1 | U |
| Sellamuthu et al., 2013 | U | U | U | U | U | U | U | U | 1 | U |
| Sellamuthu et al., 2017 | U | 1 | U | U | U | U | U | U | 1 | U |
| Shichino et al., 2015 | U | U | U | U | U | U | U | U | 1 | U |
| Shichino et al., 2019 | U | U | U | U | U | U | U | U | 1 | U |
| Song et al., 2021 | 1 | U | U | U | U | U | 1 | U | 1 | U |
| Souma et al., 2018 | U | U | U | U | U | U | U | U | 1 | U |
| Thakur et al., 2009 | U | U | U | U | U | U | U | U | 1 | U |
| Umbright et al., 2017 | U | 1 | U | U | U | 1 | U | U | 1 | U |
| Wiethoff et al., 2003 | U | 1 | U | U | U | U | U | U | 1 | U |
| Xiaojun et al., 2016 | U | 1 | U | U | U | U | U | U | 1 | U |
| Zhao et al., 2020 | 1 | U | U | U | U | 1 | U | U | 1 | U |
| Zhu et al., 2020 | 1 | 1 | U | U | U | U | U | U | 1 | U |
| **Total of « yes »** | **17** | **17** | **0** | **1** | **0** | **8** | **5** | **2** | **41** | **0** |
| Abbreviations: 1= yes ; U=Unclear ; 0 = no  Q1: Was the allocation sequence adequaly generated and applied?  Q2: Were the groups similar at baseline or were they adjusted for confounder in the analysis ?  Q3: Was the allocation to the different groups adequately concealed during ?  Q4: Were the animals randomly housed during the experiment ?  Q5: Were the caregivers and/or investigators blinded from knowledge which intervention each animal received during the experiment ?  Q6: Were animals selected at random for outcome assessment ?  Q7: Was the outcome assessor blinded?  Q8: Were incomplete outcome data adequately addressed ?  Q9: Are reports of the study free of selective outcome reporting ?  Q10: Was the study apparently free of other problems that could result in high risk of bias ? | | | | | | | | | | |
