# Supplementary material for: Results from omic approaches in rat or mouse models exposed to inhaled crystalline silica: a systematic review
Source: Part Fibre Toxicol. 2024 Mar 1;21:10. doi: 10.1186/s12989-024-00573-x (PMC10905840; doi:10.1186/s12989-024-00573-x)
Supplement: Supplementary file 4 — Additional file 4. Table S4. Summary of omics methods and organs used in studies [file 12989_2024_573_MOESM4_ESM.docx]

| **Supplementary Table 4:** Summary of omics methods and organs used in studies | | |
| --- | --- | --- |
| **Organs/fluids** | **Omics methods** | **Studies** |
| **Acute effects (≤1 week) – 10 studies** | | |
| Lung (N=9) | mRNA microarray | Sellamuthu et al., 2011 |
|  |  | Thakur et al., 2009 |
|  |  | Umbright et al., 2017 |
|  |  | Wiethoff et al., 2003 |
|  |  | Dorman et al., 2012 |
|  | NanoString nCounter | Bates et al., 2019 |
|  |  | Benninghoff et al., 2019 |
|  | SSH-cDNA sequencing | Jin et al., 2008 |
|  | miRNA microarray | Ji et al., 2015 |
|  | TOF-MS | Dorman et al., 2012 |
| Blood (N=1) | mRNA microarray | Sellamuthu et al., 2011 |
| **Sub-acute effects (2 to 11 weeks) – 30 studies** | | |
| Lung (N=25) | mRNA microarray | Beamer et al., 2010 |
|  |  | Koli et al., 2016 |
|  |  | Sai et al., 2021 |
|  |  | Sellamuthu et al., 2013 |
|  |  | Shichino et al., 2019 |
|  |  | Thakur et al., 2009 |
|  | mRNA-seq | Chen et al., 2018 |
|  |  | Pang et al., 2021 |
|  |  | Song et al., 2021 |
|  |  | Zhao et al., 2020 |
|  | NanoString nCounter | Bates et al., 2019 |
|  |  | Benninghoff et al., 2019 |
|  |  | Chauhan et al., 2021 |
|  | SSH-cDNA sequencing | Jin et al., 2008 |
|  | 3' SAGE-seq | Shichino et al., 2019 |
|  | Single cell RNA-seq | Song et al., 2021 |
|  | miRNA microarray | Faxuan et al., 2012 |
|  |  | Ji et al., 2015 |
|  | miRNA-seq | Souma et al., 2018 |
|  | lncRNA microarray | Sai et al., 2019 |
|  |  | Sai et al., 2021 |
|  | TMT LC-MS | Bo et al., 2020 |
|  | MALDI-TOF-MS | Xiaojun et al., 2016 |
|  | High-Throughput Protein Microarray Aab Profiling | Rajasinghe et al., 2020 |
|  | NMR spectroscopy | Hu et al., 2008 |
| Blood (N=1) | mRNA microarray | Sellamuthu et al., 2011 |
| Serum (N=2) | Antibody array | Cao et al., 2021 |
|  | Quantitative inflammatory microarray | Song et al., 2021 |
| Plasma (N=2) | MALDI–TOF–MS PMF | Kim et al., 2005 |
|  | High-Throughput Protein Microarray Aab Profiling | Rajasinghe et al., 2020 |
| **Long term effects (≥ 12 weeks) – 24 studies** | | |
| Lung (N=17) | mRNA Microarray | Brown et al., 2005 |
|  |  | Ellinger-Ziegelbauer et al., 2009 |
|  |  | Langley et al., 2011 |
|  |  | Sellamuthu et al., 2012 |
|  |  | Sellamuthu et al., 2013 |
|  |  | Sellamuthu et al., 2017 |
|  | mRNA-seq | Cai et al., 2021 |
|  | NanoString nCounter | Bates et al., 2019 |
|  |  | Benninghoff et al., 2019 |
|  | SSH-cDNA sequencing | Jin et al., 2008 |
|  | NGS analysis | Sager et al., 2020 |
|  | miRNA-seq | Gao et al., 2020 |
|  | lncRNA-seq | Cai et al., 2020 |
|  | iTRAQ LC-MS | Zhu et al., 2020 |
|  | NMR spectroscopy | Hu et al., 2008 |
|  | High-Throughput Protein Microarray Aab Profiling | Pestka et al., 2021 |
|  |  | Rajasinghe et al., 2020 |
| Blood (N=2) | mRNA microarray | Sellamuthu et al., 2011 |
|  |  | Sellamuthu et al., 2012 |
| Plasma (N=3) | MALDI–TOF–MS PMF | Kim et al., 2005 |
|  | High-Throughput Protein Microarray Aab Profiling | Pestka et al., 2021 |
|  |  | Rajasinghe et al., 2020 |
| Spleen and kidney (N=2) | NanoString nCounter | Bates et al., 2019 |
|  |  | Benninghoff et al., 2019 |
