# Supplementary figures and images for: Results from omic approaches in rat or mouse models exposed to inhaled crystalline silica: a systematic review
Source: Part Fibre Toxicol. 2024 Mar 1;21:10. doi: 10.1186/s12989-024-00573-x (PMC10905840; doi:10.1186/s12989-024-00573-x)

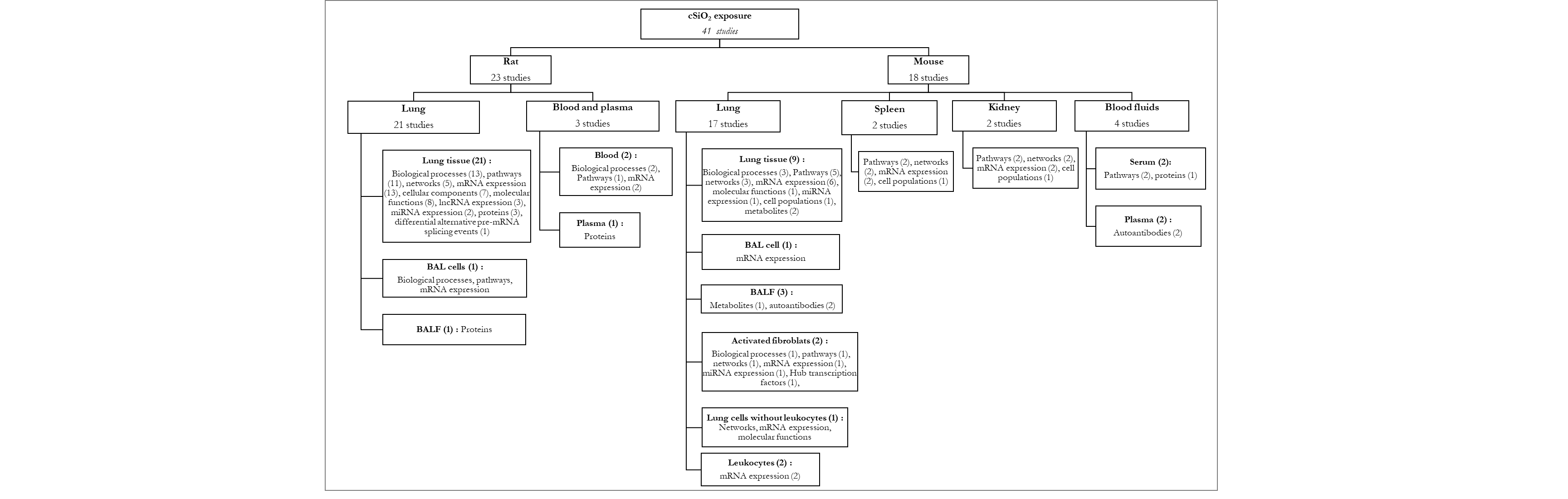


**Supplementary Figure 1:** Crystalline silica exposure outcomes studied in rat and mouse organs

Supplement: Supplementary file 5 — Additional file 5. Fig. S1. Crystalline silica exposure outcomes studied in rat and mouse organs [file 12989_2024_573_MOESM5_ESM.docx]
