# Supplementary material for: Results from omic approaches in rat or mouse models exposed to inhaled crystalline silica: a systematic review
Source: Part Fibre Toxicol. 2024 Mar 1;21:10. doi: 10.1186/s12989-024-00573-x (PMC10905840; doi:10.1186/s12989-024-00573-x)
Supplement: Supplementary file 6 — Additional file 6. Fig. S2. Heatmap representing the main cellular responses sub-domains found in the lungs or at systemic level (serum, plasma, spleen) in included studies. Heatmap is expressed as the percentage of cellular response sub-domains of all biological processes, pathways and networks mentioned in studies among. the total number of different cellular response terms in lungs (N = 61) and at systemic level (N = 2) retrieved in all included studies. [file 12989_2024_573_MOESM6_ESM.docx]

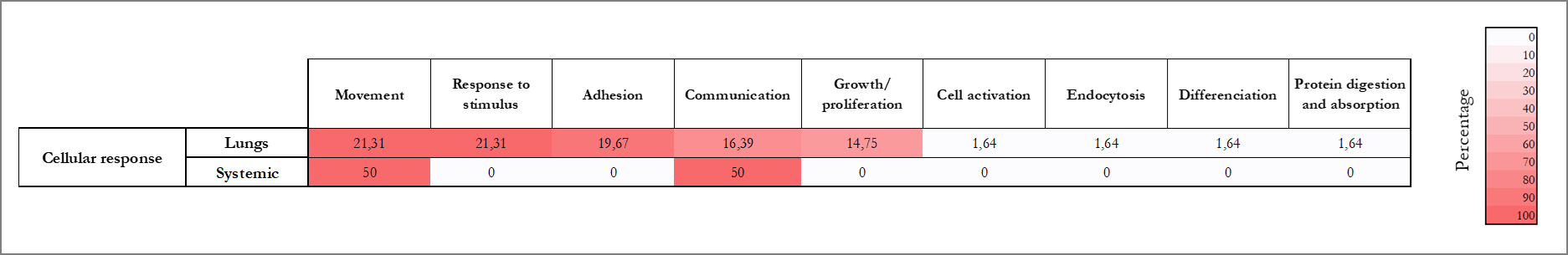


**Supplementary Figure 2: Heatmap representing the main cellular responses sub-domains found in the lungs or at systemic level (serum, plasma, spleen) in included studies.** Heatmap is expressed as the percentage of cellular response sub-domains of all biological processes, pathways and networks mentioned in studies among. the total number of different cellular response terms in lungs (N=61) and at systemic level (N=2) retrieved in all included studies.
